# Decreased serum superoxide dismutase concentration has a high value for the diagnosis of periprosthetic joint infection—a single-center, retrospective study

**DOI:** 10.1186/s12891-022-05965-8

**Published:** 2022-11-21

**Authors:** Shuo Yan, Xiaofei Zhang, Zhen Lyu, Jun Liu

**Affiliations:** 1 Department of Joints, Tianjin Hospital, No. 406 Jiefang South Rd, Tianjin, 300211 Hexi District China; 2 Graduate School of Tianjin Medical University, 22 Qixiang Tai Road, Tianjin, 300203 Heping District China; 3 Department of Orthopaedics, Tianjin Medical University General Hospital, Anshan Road 154, Tianjin, 300052 Heping District China

**Keywords:** Periprosthetic joint infection, Total joint arthroplasty, Superoxide dismutase, Diagnosis, Biomarker

## Abstract

**Purpose:**

As the most serious complication of total knee arthroplasty (TKA), periprosthetic joint infection (PJI) often leads to disastrous consequences. An accurate preoperative diagnosis plays a significant role in saving prostheses and optimizing treatment outcomes. Through this retrospective case–control study, we aimed to investigate the potential of superoxide dismutase (SOD) as a novel serum biomarker in the diagnosis of PJI.

**Methods:**

We conducted a retrospective review of all patients who underwent TKA and received adequate follow-ups in our hospital from June 2015 to December 2021. A total of 50 patients were enrolled in the PJI group based on the 2018 International Consensus Meeting (ICM) criteria. Besides that, we enrolled 100 patients who underwent TKA in the same period and had a good postoperative course in the control group. Patient characteristics, comorbidities, laboratory results (serum, synovial, and microbial), and intraoperative findings (purulence and histopathology) were documented and compared by univariate analysis. Receiver operating characteristic (ROC) analysis was used to determine the sensitivity, specificity, and diagnostic performance.

**Results:**

The median serum SOD level in the PJI and control group was 135.95 ± 24.47 U/ml (IQR, 111.85–158.30 U/ml) and 173.83 ± 13.9 U/ml (IQR,162.83–183.5 U/ml) (*p* < 0.05), respectively. With the calculated cutoff of SOD at 149.5U/L, the area under the ROC curve (AUC), sensitivity, specificity, positive predictive value (PPV), and negative predictive value (NPV), were 0.919, 0.72, 0.97, 0.923, and 0.874, respectively. In subgroup analysis, the specificity of SOD in diagnosing culture-negative PJI remained extremely high (0.98). Combined diagnosis of serum SOD and C-reactive protein (CRP) made AUC increase to 0.972.

**Conclusion:**

Serum SOD showed great potential in the diagnosis of PJI.

## Introduction

Over the past half-century, total knee arthroplasty (TKA) has become one of the revolutionary advances in medicine, which can effectively relieve the pain of the patients with end-stage osteoarthritis and improve the life quality. As the most serious complication of TKA, postoperative periprosthetic joint infection (PJI) often leads to disastrous consequences, which not only bring great inconvenience to the lives of patients but also impose an enormous financial burden on the entire medical system [1–3]. Thus, in order to avoid missing the optimal antibiotic prophylaxis and debridement time, an accurate preoperative diagnosis plays a significant role in saving prostheses and optimizing treatment outcomes.

With the deepening of people’s understanding of PJI in recent years, the diagnostic methods and standards have been updated. The diagnostic criteria proposed in the 2018 International Consensus Meeting (ICM) on Musculoskeletal Infection has been widely recognized by clinicians all over the world [4]. Guidelines recommend that the accurate diagnosis of PJI should be comprehensively considered from four aspects: clinical characteristics, serum and synovial fluid biomarkers, microbial cultivation and histological examination.

Serum biomarkers are the first choice for diagnosing PJI. In addition to the traditional inflammatory biomarker CRP and erythrocyte sedimentation rate (ESR), some novel serum biomarkers including fibrinogen and D-dimer have shown excellent diagnostic values. But the sensitivity and specificity still had limitations [5–8]. Normal laboratory results are common in clinical work, especially if the PJI is present in the form of a low-grade or chronic encapsulated infection [9, 10].

In recent years, some synovial fluid biomarkers, such as white blood cell count (WBC), Polymorphonuclear% (PMN %), C-reactive protein (CRP), α-defensin, and leukocyte esterase (LE) have been regarded as effective supplements to serum inflammatory biomarkers [11]. A meta-analysis involving multiple synovial biomarkers pointed out that biochemical examination of synovial fluid has important diagnostic value (with sensitivity ≥ 0.8 and specificity ≥ 0.9). But the articular puncture is an invasive operation, which could possibly cause iatrogenic infection. Moreover, it is usually difficult to obtain sufficient joint synovial fluid to meet the needs of examination, resulting in false-negative results [12].

The microbial cultivation is also less effective than expected because it will lead to false-negative results due to antibiotic use. About 12% of culture-negative cases contain bacteria when tested by the latest molecular biological methods [13]. Besides that, the error which caused by operational pollution cannot be ignored [14]. Conditioned pathogens on the surface of the skin may appear in the sample, resulting in false positive results.

For some suspected PJI cases, it is still difficult to make an accurate preoperative diagnosis on existing laboratory biomarkers. The diagnosis largely based on intraoperative conditions and postoperative histological results. Orthopedic scholars have been trying to find more ideal laboratory biomarkers to compensate for the preoperative diagnostic evidence [15, 16].

Some studies found the occurrence of inflammatory diseases is often accompanied by the changed activity of superoxide dismutase (SOD) [17, 18]. SOD plays a crucial etiopathology role in inflammatory diseases by catalyzing the conversion of superoxide to hydrogen peroxide and oxygen [19, 20]. The significant decrease in serum SOD level can be regarded as the weakened anti-inflammatory and antioxidant capacity of the body. Thus, in regard to the diagnosis of PJI, serum SOD has potential research significance.

Through this retrospective case–control study, we aimed to investigate the potential of SOD as a novel serum biomarker in diagnosis of PJI and calculated the optimal threshold.

## Materials and methods

After the institutional review board approved and waived the need to obtain informed consent, we reviewed the electronic medical record system of Tianjin Hospital and eventually completed a single-center, retrospective cohort study. The database covered all cases of knee arthroplasties and revisions performed in our hospital from June 2015 to December 2021. The information of patients in the database was kept confidential for the purpose of protecting participants’ privacy, and all methods were carried out in accordance with relevant guidelines.

Patients who were scheduled to have revision surgery due to PJI were enrolled in the case group. Besides that, we selected patients who underwent TKA in the same period and had a good postoperative course in the control group according to the ratio of 1:2. The diagnosis of PJI was based on the 2018 ICM criteria (Table 1) [4].

**Table 1:** The 2018 International Consensus Meeting (ICM) definition of PJI

Major Criteria		Diagnosis
Two positive cultures of the same organism		Infected
Sinus tract communicating with the joint or visualization of the implant		
Minor Criteria (Preoperative Diagnosis)	Score	Diagnosis
Elevated CRP (100 mg /L) or Dimer (860 ng /ml)	2	≥ 6 Infected 2–5 Possibly Infected 0–1 Not Infected
Elevated ESR (30 mm /h)	1	
Elevated synovial WBC count(3000//μl) or leukocyte esterase (+ +)	3	
positive alpha-defensin	3	
Elevated synovial PMN percentage (80%)	2	
Elevated synovial CRP (6.9 mg /L)	1	
Inconclusive pre-op score or dry tap (Intraoperative Diagnosis)	Score	
Preoperative score	-	≥ 6 Infected 4–5 Inconclusive ≤ 3 Not Infected
Positive histology	3	
Positive purulence	3	
Single positive culture	2	

*PJI* periprosthetic joint infection, *CRP* C-reactive protein, *ESR* erythrocyte sedimentation rate, *LE* leukocyte esterase, *PMN* polymorphonuclear, *WBC* white blood cell

Before revisions, we made a comprehensive evaluation of the patients according to imaging examination, clinical manifestations, serum and synovial fluid biomarkers, and the results of microbial cultivation. During operations, we first observed whether there was purulent fluid around the prosthesis and whether there was a hidden sinus in the articular cavity. Then we collected tissue samples from the medullary cavity or prosthesis-bone interface for microbial cultivation and drug sensitivity tests. Finally, we sent the lesion specimen for pathological examination after surgery. The final diagnosis of PJI was based on the scores of two independent researchers, and if the conclusions were inconsistent, the final judgment was made by the correspond author.

The exclusion criteria are as follows.

- (1). Patients who were definitely diagnosed with aseptic loosening (AL). (The definition of AL was based on whether there was locally progressive pain and joint dysfunction following TKA, whether a translucent band with a diameter > 2 mm around the loose prosthesis could be observed by imaging examination, and whether the inflammatory biomarkers were negative [21].)
- (2).Patients with skin ulcers, hematoma, or other orthopedic infectious diseases.
- (3).Patients combined with autoimmune diseases, such as rheumatoid arthritis (RA), ulcerative colitis (UC), systemic lupus erythematosus (SLE).
- (4).Patients with malignancy or with severe liver or kidney dysfunction.
- (5).Patients with hematological diseases or receiving anticoagulant therapy.
- (6).Patients with incomplete records of laboratory information.

**Fig. 1:**
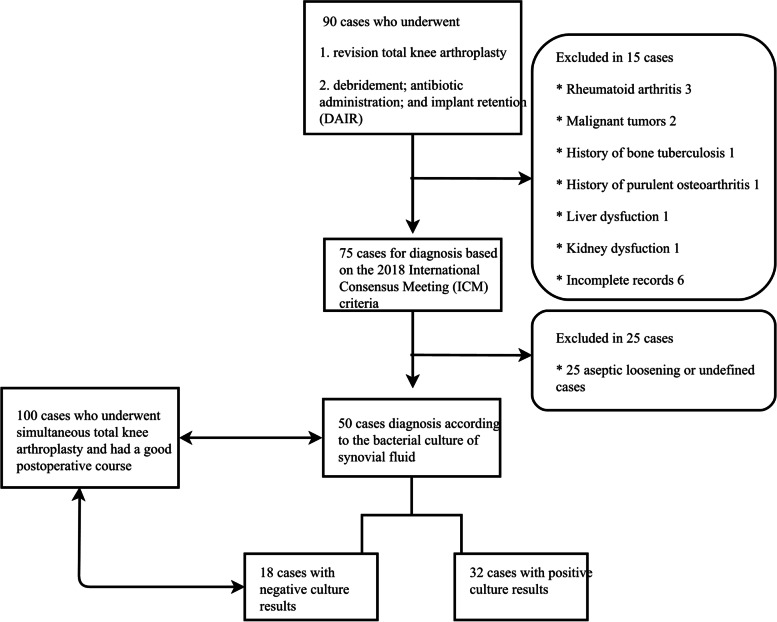
Flowchart of the included and excluded patients

Exclusion criteria from (2) to (5) was considered because such diseases may affect the levels of inflammatory biomarkers, thereby interfering with the diagnostic value. The flowchart of the included and excluded patients was presented in Fig. 1.

Patient characteristics, comorbidities, laboratory results (serum, synovial, and microbial), and intraoperative findings (purulence and histopathology) were documented and compared by univariate analysis. All patients’ peripheral venous blood samples were collected on the morning of the first day after admission and were sent to the laboratory of Tianjin Hospital for testing. SOD was measured by the pyrogallol autooxidation method, and the operation steps were strictly in accordance with the standards. The SOD reference value provided by the manufacturer ranged from 129 U/ml to 216 U/ml.

### Statistical analysis

All of the statistical analyses were performed with the IBM SPSS Statistics software (version 28). Means and standard deviations were described for quantitative data and frequencies were described for qualitative data. To evaluate baseline characteristics, categorical variables were summarized using chi-squared tests, whereas the continuous variables between the 2 groups were compared with the Mann–Whitney test and a *P* < 0.05 was considered statistically significant. The receiver operating characteristic (ROC) curve was analyzed by MedCalc statistical software (version 19). The sensitivity, specificity, positive predictive value (PPV), negative predictive value (NPV), and the area under the ROC curve (AUC) were calculated and recorded in a dedicated statistical datasheet (Microsoft Excel). AUC can range between 0 and 1. The value of AUC was interpreted as excellent (0.90–1.00), good (0.80–0.89), fair (0.70–0.79), poor (0.60–0.69), or failing (0.50–0.59). The Youden index was used to determine the optimal cutoff of each biomarker. The boxplot was drawn by GraphPad Prism software (version 8).

## Results

### Clinical characteristics of the enrolled patients

Finally, the patients enrolled in this study were divided into the PJI group (*n* = 50) and the non-PJI group (*n* = 100). In the PJI group, the patients were divided into the culture-negative group (*n* = 18) and the culture-positive group (*n* = 32) according to the results of microbial cultivation. There was no significant difference in age, BMI, and comorbidities between the two groups except for gender.

### Microbiology data

**Fig. 2:**
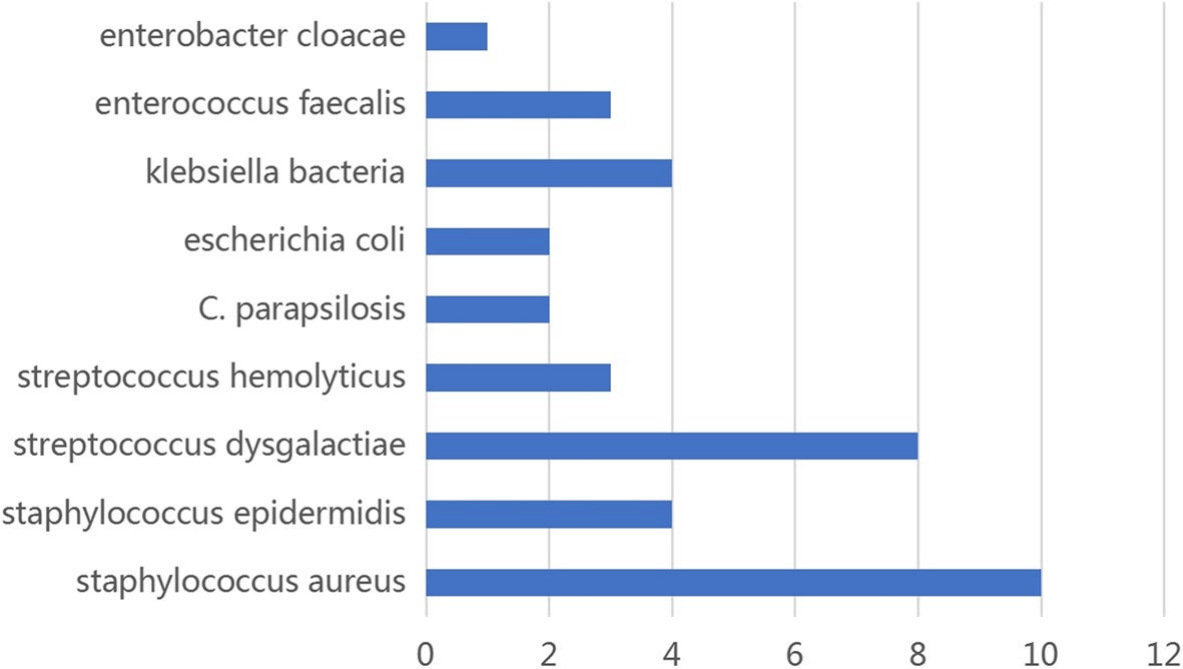
The distribution of cultural results

A total of 37 strains of bacteria or fungi were cultured from 32 patients. Staphylococcus aureus and Staphylococcus dysgalactiae were the most common we observed. The distribution of cultural results was presented in Fig. 2.

### Preoperative serum biomarkers of the enrolled patients

In the comparison of PJI and non-PJI group, the mean number of serum CRP was 61.84 ± 43.04 mg/L (interquartile range [IQR], 15.50–105.25 mg/L) and 7.39 ± 9.10 mg/L (IQR,5.00–5.00 mg/L) (*p* < 0.05), respectively, while serum SOD was 135.95 ± 24.47 U/ml (IQR, 111.85–158.30 U/ml) and 173.83 ± 13.9 U/ml (IQR,162.83–183.5 U/ml) (*p* < 0.05), respectively. (Table 2).

**Table 2:** Demographic characteristics of the enrolled patients

		**Group A (50)**	**Group B (100)**	***P*-value**
**Age**		67.48 ± 8.00	66.29 ± 6.33	NS
**Sex**				*P* < 0.05
	Male	25	23	
	Female	25	77	
**BMI**		27.32 ± 3.61	27.90 ± 3.93	NS
**Affected joint**	Left Knee	24	46	NS
	Right Knee	26	54	NS
**Comorbidities**	Hypertension	32	61	NS
	Diabetes mellitus	13	15	NS
	Cardiovascular disease	9	15	NS
	Cerebrovascular disease	5	2	NS
**Preoperative bacterial culture**	positive	32		
	negative	18		
**CRP** (mg/L)		61.84 ± 43.04 mg/L	7.39 ± 9.10 mg/L	*P* < 0.05
**SOD** (U/ml)		135.95 ± 24.47 U/ml	173.83 ± 13.9 U/ml	*P* < 0.05
**WBC** (10^9^/L)		8.82 ± 4.46	6.06 ± 1.51	*P* < 0.05
**NEUT** (%)		71.02 ± 11.76	62.93 ± 6.65	*P* < 0.05
**PLT** (10^9^/L)		297.58 ± 99.51	294.39 ± 246.08	*P* = 0.206
**MPV**(FL)		9.73 ± 0.93	10.38 ± 0.74	*P* < 0.05
**PC/MPV**		31.30 ± 11.53	28.74 ± 24.44	*P* < 0.05

Notes: *CRP* C-reactive protein, *WBC* white blood cell, *NEUT%* Neutrophil percentage, *PLT* platelet count, *MPV* Mean platelet volume, *SOD* superoxide dismutase

The *p*-value indicates statistical significance, *p* < 0.05

**Fig. 3:**
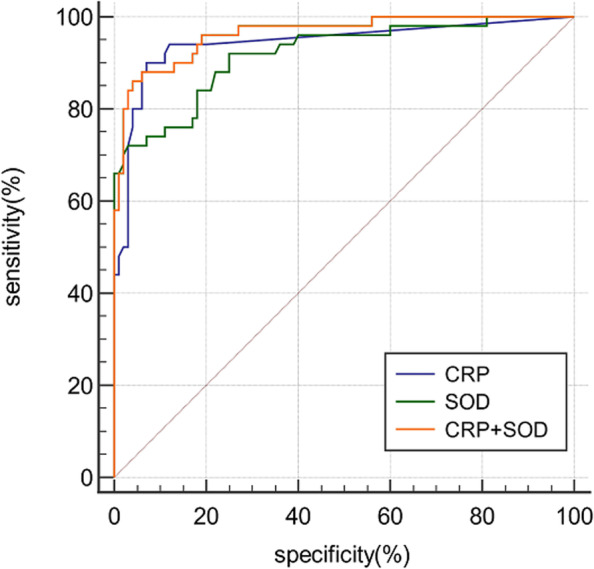
Receiver operating characteristic (ROC) curves show the predictive value of SOD and CRP of PJI. AUC can range between 0 and 1. The closer the curve is located in the upper-left-hand corner and the larger the AUC, the better value is in the diagnosis of PJI

Figure 3 and Table 3 described the diagnostic value of each biomarker. With the calculated cutoff of CRP at 11.5 mg/L, the AUC, sensitivity, specificity, PPV, and NPV were 0.943, 0.9, 0.93, 0.865, and 0.949, respectively. While with the calculated cutoff of SOD at 149.5U/L, the AUC, sensitivity, specificity, PPV, and NPV were 0.919, 0.72, 0.97, 0.923, and 0.874, respectively. Other laboratory biomarkers were excluded from the next discussion because ROC analysis showed no excellent diagnostic value. The combinations of serum SOD and CRP led to the improvement in AUC and specificity, however, at the expense of a decrease in sensitivity (AUC, sensitivity, specificity, PPV, and NPV were 0.963, 0.88, 0.94, 0.88, and 0.94, respectively).

**Table 3:** Results of the ROC Curve Analysis on serum SOD and CRP Between PJI group and non-PJI group

	**AUC**	**95%CI**	**Optimal cutoff**	**Youden index**	**Sensitivity**	**Specificity**	**PPV**	**NPV**	***P*-Value**
CRP	0.943	0.893—0.974	> 11 mg/L	0.83	90	93	86.5	94.9	< 0.0001
SOD	0.919	0.863—0.957	≤ 149.5U/ml	0.69	72	97	92.3	87.4	< 0.0001
CRP + SOD	0.963	0.919–0.987	——	0.82	88	94	88	94	< 0.0001

*CRP* C-reactive protein, *SOD* superoxide dismutase, *PPV* Positive Predictive Value, *NPV* Negative Predictive Value, *LR* + positive likelihood ratio, *LR − *negative likelihood ratio

### Preoperative serum SOD and CRP in the subgroup analysis

**Fig. 4:**
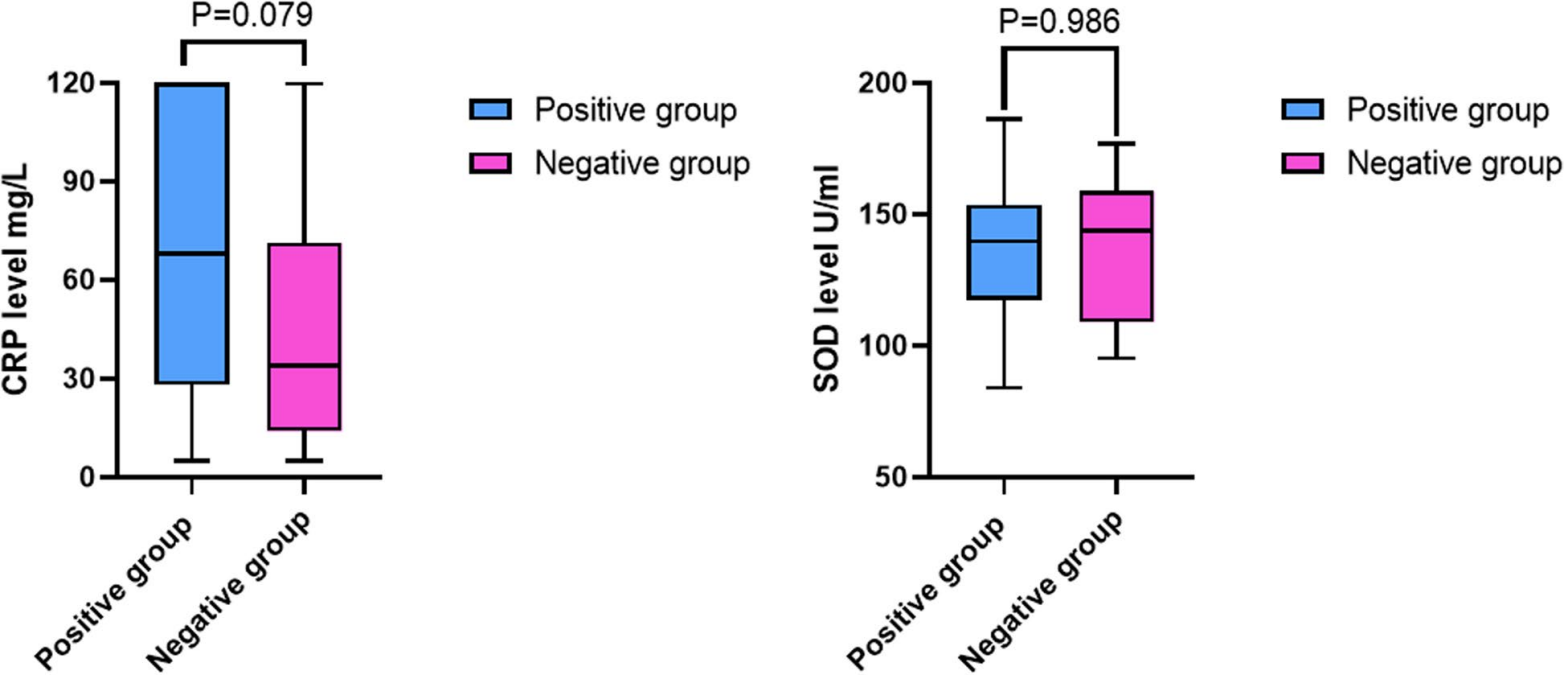
Boxplots showing the distribution of CRP (left) and SOD (right) between the culture-positive group (*n* = 32) and culture-negative group (*n* = 18). The horizontal line represents the median level, the upper and lower lines represent the interquartile range

**Fig. 5:**
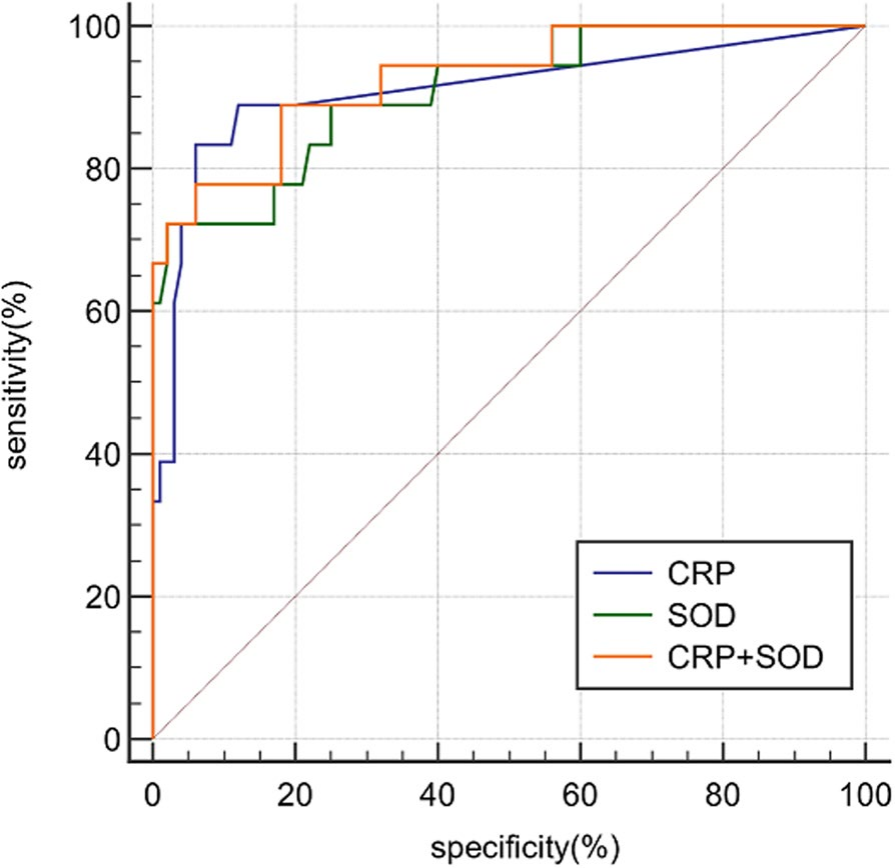
Receiver operating characteristic (ROC) curves show the predictive value of SOD and CRP of culture-negative PJI

In the subgroup analysis of the culture-negative group versus the culture-positive group, the change of SOD (136.0 ± 24.03 U/ml VS 135.9 ± 25.96 U/ml) was not as obvious as that of CRP (69.84 ± 43.15 mg/L VS 47.61 ± 40.11 mg/L). (Fig. 4) For diagnosing PJI with negative-culture results, SOD showed an AUC of 0.908 with an optimal cutoff point of 149 U/ml. The sensitivity and specificity were 0.72 and 0.98, respectively. With the optimal cutoff point of 13 mg/L, CRP had an AUC of 0.909, a sensitivity of 0.83 and a specificity of 0.90. Combined diagnosis of serum SOD and CRP made AUC increase to 0.972. (Fig. 5 + Table 4).

**Table 4:** Results of the ROC Curve Analysis on serum SOD and CRP Between culture-negative PJI group and non-PJI group

	**AUC**	**95%CI**	**Optimal cutoff**	**Youden index**	**Sensitivity**	**Specificity**	**PPV**	**NPV**	***P*-Value**
CRP	0.909	0.842—0.954	> 13 mg/L	0.77	83.3	90	83	93	< 0.0001
SOD	0.908	0.840—0.953	≤ 149U/ml	0.7	72.2	98	72	98	< 0.0001
CRP + SOD	0.972	0.864–0.967	——	0.72	77.8	94	78	98	< 0.0001

*CRP* C-reactive protein, *SOD* superoxide dismutase, *PPV* Positive Predictive Value, *NPV* Negative Predictive Value, *LR* + positive likelihood ratio, *LR − *negative likelihood ratio

## Discussion

Alterations in the expression level of serum SOD have been found in a variety of pathological conditions, including diabetes mellitus, autoimmune diseases, as well as infectious diseases [20, 22–26]. A previous study reported that SODs can be used to treat oxidative stress-associated inflammatory diseases [27]. Furthermore, some scholars also confirmed that serum SOD could be a useful prediction marker in infection-related adverse events. For instance, according to a Chinese scholar 's research report, the plasma SOD levels were significantly decreased in the pneumonia group and sepsis group. Lin, S. P. et al*.* demonstrated that serum SOD can be served as a new indicator of infection in ischemic stroke patients [28]. Another research also showed that SOD activity was highly accurate in predicting adverse events at the early stage of acute pancreatitis. [29].

To our knowledge, this is the first retrospective case–control study to evaluate the potential of serum SOD as a novel diagnostic biomarker for PJI. We found that serum SOD level was lower in PJI patients and the diagnostic value was highest with the calculated cutoff of 149.5U/L.

The inflammatory response of the body is a complex response to infection. In recent years, a large number of serum inflammatory biomarkers have been studied for the diagnosis of PJI. The promising serum inflammatory biomarkers include IL-4, IL-6, TNF-α, and procalcitonin [30]. These inflammatory cytokines are secreted by various inflammatory cells.

SOD can inhibit inflammatory response by eliminating the reactive oxygen species (ROS), regulating immune cell function (T cells, macrophages, NK cells, and dendritic cells), inhibiting inflammatory mediators, and regulating cell signaling cascades (TLRs, NF-κB, MAPKs, and JAK-STAT) [20]. When the pathogenic bacteria settled on the prosthetic surface and caused inflammatory reactions with the prolongation of postoperative time, the significant decrease in serum SOD can be regarded as a protective mechanism. More importantly, it seems that the serum SOD level does not fluctuate greatly due to the change in various bacterial virulence. When CRP is used alone to diagnose culture-negative PJI, the specificity is relatively poor. The combination of SOD and CRP may make up for this shortcoming.

Our study has some limitations. Firstly, this study is a retrospective single-center study consisting of only a small number of cases. The main reason for the small sample size is the low incidence of PJI. Large-scale multi-center data collection may make the research results more reliable. Secondly, there is no gold standard in the strict sense for diagnosing PJI at present. It may cause some selection bias, resulting in some patients with special PJI being excluded. Thirdly, preoperative use of antibiotics may affect the results of various tests. Fourthly, the low feasibility of conducting all diagnostic tests remains a major clinical concern. For instance, some synovial fluid biomarkers were not completely collected due to the limitation of our laboratory testing capability and insufficient synovial fluid sample. Although several synovial parameters which served as secondary criteria were missing, such PJI patients had sufficient scores to meet PJI diagnosis according to the ICM criteria.

## Conclusion

Serum SOD showed excellent performance in the diagnosis of PJI. Specifically, it can improve the diagnostic value combined with CRP for culture-negative PJI. As a laboratory examination with strong maneuverability and high accuracy, serum SOD is expected to be used in future clinical work. More high-quality prospective randomized controlled trials (RCTs) are needed to validate the diagnostic value of serum SOD.

## Data Availability

The datasets used during the current study available from the corresponding author on reasonable request.

## Abbreviations

TKA: Total knee arthroplasty
AL: Aseptic loosening
PJI: Periprosthetic joint infection
SOD: Superoxide dismutase
CRP: C-reactive protein
WBC: White blood cell count
PMN%: Polymorphonuclear%
LE: Leukocyte esterase
PC: Platelet count
MPV: Platelet count
ESR: Erythrocyte sedimentation rate;
ICM: International Consensus Meeting
ROC: Receiver operating characteristic
AUC: The area under the ROC curve
PPV: Positive predictive value
NPV: Positive predictive value
RA: Rheumatoid arthritis
UC: Ulcerative colitis
SLE: Systemic lupus erythematosus
SAI: Stroke-associated infection
RCTs: Randomized controlled trials
